# Identification of Anti-Neuroinflammatory Bioactive Compounds in Essential Oils and Aqueous Distillation Residues Obtained from Commercial Varieties of *Cannabis sativa* L.

**DOI:** 10.3390/ijms242316601

**Published:** 2023-11-22

**Authors:** Maria Cristina Barbalace, Michela Freschi, Irene Rinaldi, Eugenia Mazzara, Tullia Maraldi, Marco Malaguti, Cecilia Prata, Filippo Maggi, Riccardo Petrelli, Silvana Hrelia, Cristina Angeloni

**Affiliations:** 1 Department for Life Quality Studies, Alma Mater Studiorum-University of Bologna, Corso D’Augusto 237, 47921 Rimini, Italy; maria.barbalace2@unibo.it (M.C.B.); michela.freschi2@unibo.it (M.F.); irene.rinaldi5@unibo.it (I.R.);; 2 Chemistry Interdisciplinary Project (ChIP) Research Center, School of Pharmacy, University of Camerino, Via Madonna delle Carceri, 62032 Camerino, Italy; eugenia.mazzara@unicam.it (E.M.); filippo.maggi@unicam.it (F.M.); riccardo.petrelli@unicam.it (R.P.); 3 Department of Biomedical, Metabolic and Neural Sciences, University of Modena and Reggio Emilia, Via del Pozzo 71, 41125 Modena, Italy; tullia.maraldi@unimore.it; 4 Department of Pharmacy and Biotechnology, Alma Mater Studiorum-University of Bologna, Via Irnerio, 48, 40126 Bologna, Italy; cecilia.prata@unibo.it

**Keywords:** *Cannabis*, microglia, neuroinflammation, anti-inflammatory activity, essential oils, aqueous distillation residues

## Abstract

Neuroinflammation, which is mainly triggered by microglia, is a key contributor to multiple neurodegenerative diseases. Natural products, and in particular *Cannabis sativa* L., due to its richness in phytochemical components, represent ideal candidates to counteract neuroinflammation. We previously characterized different *C. sativa* commercial varieties which showed significantly different chemical profiles. On these bases, the aim of this study was to evaluate essential oils and aqueous distillation residues from the inflorescences of three different hemp varieties for their anti-neuroinflammatory activity in BV-2 microglial cells. Cells were pretreated with aqueous residues or essential oils and then activated with LPS. Unlike essential oils, aqueous residues showed negligible effects in terms of anti-inflammatory activity. Among the essential oils, the one obtained from ‘Gorilla Glue’ was the most effective in inhibiting pro-inflammatory mediators and in upregulating anti-inflammatory ones through the modulation of the p38 MAPK/NF-κB pathway. Moreover, the sesquiterpenes (*E*)-caryophyllene, α-humulene, and caryophyllene oxide were identified as the main contributors to the essential oils’ anti-inflammatory activity. To our knowledge, the anti-neuroinflammatory activity of α-humulene has not been previously described. In conclusion, our work shows that *C. sativa* essential oils characterized by high levels of sesquiterpenes can be promising candidates in the prevention/counteraction of neuroinflammation.

## 1. Introduction

Neuroinflammation plays a key role in the pathophysiological process of various neurodegenerative diseases and neurological disorders [1,2,3]. This process involves a coordinated response between microglia and other central nervous system (CNS) cells, such as astrocytes [4]. A crucial role is played by microglial cells, the resident immune cells of the CNS, which are essential for brain homeostasis and development [5]. Microglia act as the defense mechanism for the brain, particularly in response to injuries like infections and stroke; however, it is worth noting that activated microglia can exhibit dual effects.

In pathological circumstances, microglia can indeed become hyperactivated, resulting in neuronal toxicity and neuronal damage [6]. During activation, microglial cells can exist in two different phenotypes: the M1 phenotype (classical), pro-inflammatory, is associated with the release of inflammatory mediators like tumor necrosis factor alpha (TNF-α), interleukine-1β (IL-1β), and IL-6, reactive oxygen species (ROS) and with the up-regulation of inducible nitric oxide synthase (iNOS), cyclooxygenase-2 (COX-2) [7,8]; the M2 phenotype (alternative), anti-inflammatory, exerts neuroprotective activity ameliorating neuroinflammation through the increase of anti-inflammatory mediators and growth factors, like IL-10, IL-4, transforming growth factor-β (TGF-β), arginase-1 (Arg-1), and mannose receptor C-type 1 (MRC1) [8,9]. Once activated, M1 and M2 cells can coexist as single or mixed phenotypes, underlying the complexity in the microglia dynamics [10]. Furthermore, mounting evidence has demonstrated an association between the release of pro-inflammatory cytokines from microglia and the activation of nucleotide-binding and oligomerization domain (NOD)-like receptor family, pyrin domain containing-3 protein (NLRP3) inflammasome [11]. The NLRP3 represents a multi-protein complex that, once assembled and activated, induces proteolytic cleavages and maturation of pro-inflammatory cytokines such as IL-1β and IL-18. These detrimental effects resulting from hyperactivated microglia have been linked to various neurodegenerative disorders, including Alzheimer’s disease (AD), Parkinson’s disease (PD), multiple sclerosis, schizophrenia, traumatic brain injury, and stroke [12,13,14]. Therefore, the simultaneous deactivation of the M1 phenotype and the induction of the protective M2 phenotype seem to be a promising strategy to counteract neuroinflammation-related pathologies.

In recent years, research has increasingly focused on medicinal plants as abundant sources of phytochemicals with promising neuroprotective efficacy. This is particularly relevant due to the current absence of drugs capable of halting or slowing down the progression of many neurodegenerative diseases.

The non-psychotropic *Cannabis sativa* L., commonly known as industrial hemp, has recently attracted attention for its pharmacological applications due to the presence of various secondary metabolites with biological activity significant for human health [15]. Essential oils (EOs) derived from hemp inflorescences are yellow liquids rich in different bioactive molecules, including mostly monoterpenes and sesquiterpenes, and sometimes also cannabinoids [16,17]. Hemp EOs have been associated with various biological activities, such as insecticidal, antimicrobial, fungicidal, antioxidant, inhibition of acetyl-cholinesterase, and neuroprotective activities [18,19,20,21]. Despite their many and intriguing biological properties, terpenes from hemp EOs have not been extensively studied. Terpenes are mainly secreted in hemp inflorescences, giving the plant its characteristic aroma, and in the form of mono- and sesquiterpenes represent the main phytochemicals of hemp EOs. Recent investigations have demonstrated significant health benefits associated with terpenes, including anti-neuroinflammatory, antioxidant, analgesic, anxiolytic, anti-bacterial, and anti-fungal activities [15,22,23]. Another underexplored fraction of hemp phytochemicals is represented by phenolic compounds, which are mainly present in the aqueous residue of inflorescences produced during distillation [24]. Among hemp phenolics, some of them, like cannflavin A, B and C, are exclusive of *C. sativa*, and have been shown to possess many beneficial properties including but not limited to anti-inflammatory, anti-cancer activities [15].

The phytochemical composition of hemp extracts and EOs is influenced by several factors, like cultivars, cultivation site, harvest time, and sample treatments [25,26].

It is also important to underline the so-called ‘entourage effect’ described for the hemp phytocomplex, due to the co-presence of terpenes, phenolic compounds, and cannabinoids which can interact with each other, leading to a synergistic effect not replicable by a singular component [27]. In this context, increasing knowledge of the composition and biological activities of hemp extracts and EOs obtained from different hemp varieties or cultivars may be advantageous.

Many hemp varieties are cultivated in Italy to satisfy the requests of different sectors, such as the textile, paper, composite materials, food, and cosmetics industries. In a recent paper we investigated the phytochemistry of nine commercial varieties which are cultivated in central Italy and used to produce craft beers [28]. Among them, the ‘White Shark’, ‘Gorilla Glue’, and ‘Lemon Conti Kush New’ varieties showed significantly different chemical profiles, in terms of their main volatile terpenes and phenolic compounds, deserving further biological investigations in order to understand the potential role of the different chemical fractions in the prevention of neuroinflammation.

Given these premises, the objective of this study was to explore the anti-neuroinflammatory activity of EOs and aqueous distillation residues (ARs) derived from the inflorescences of the three aforementioned hemp varieties in BV-2 microglial cells activated with lipopolysaccharide (LPS), to establish a neuroinflammation model.

## 2. Results

### 2.1. Chemical Constituents of Hemp EOs and ARs

The chemical profiles of the three *C. sativa* EOs and ARs investigated in this work are summarized in Table 1 and have previously been reported by Mazzara et al. [28]. Briefly, the EO of White Shark (WS) was characterized by monoterpenes such as myrcene (20.3%,) and α-pinene (8.5%), while that of Lemon Conti Kush New (LKN) was characterized by terpinolene (30.5%) and myrcene (11.1%). On the other hand, Gorilla Glue (GG) EO contained higher levels of sesquiterpenes such as (*E*)-caryophyllene (18.9%), caryophyllene oxide (6.6%) and α-humulene (4.9%).

The ARs were devoid of cannabinoids and contained mostly flavone glycosides such as apigenin and luteolin derivatives. WS extract was characterized by rutin (7.93 mg/g) and apigenin-hexoside-glucuronide (1.12 mg/g), LKN extract contained high amounts of luteolin-hexoside-hexoside (2.35 mg/g), while the AR of GG showed luteolin-hexoside-rutinoside (2.51 mg/g) as the main constituent.

### 2.2. Potential Cytotoxicity of Hemp EOs and ARs

**Figure 1:**
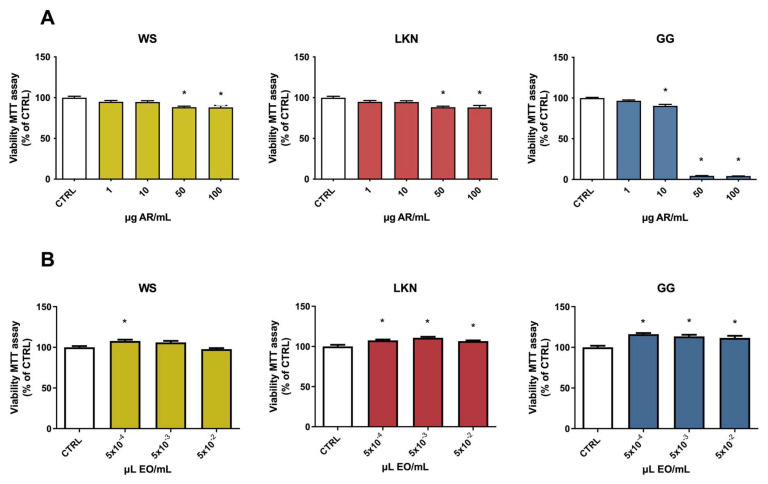
Viability of BV-2 cells treated with different concentrations of three hemp aqueous residues (ARs) and essential oils (EOs). BV-2 cells were exposed to increasing concentrations of White Shark (WS), Gorilla Glue (GG) and Lemon Conti Kush New (LKN) ARs (1–10–50–100 µg/mL) (**A**) and EOs (5 × 10^−4^–5 × 10^−3^–5 × 10^−2^ µL/mL) (**B**) for 24 h and cell viability was measured by MTT. Data are represented as % of CTRL. Each bar represents means ± SEM of at least three independent experiments. Data were analyzed by one-way ANOVA followed by Dunnett’s test. * *p* < 0.05 vs. CTRL.

BV-2 cells were treated with increasing concentrations of WS, LKN, and GG ARs and EOs to investigate their potential cytotoxicity. All the tested ARs showed cytotoxicity but with different profiles (Figure 1A). GG extract showed cytotoxicity starting from 10 µg/mL, with a stronger effect at 50 and 100 µg/mL. The other extracts significantly reduced cell viability only from 50 µg/mL, with a milder effect in respect to GG. On these bases, concentrations lower than 10 µg/mL were used in the next experiments.

On the other hand, none of the tested EOs showed cytotoxicity in BV-2 cells. In fact, the cell viability of EO-treated cells was comparable to control cells at any tested concentration (Figure 1B). Interestingly, WS, LKN, and GG EOs induced a slight but significant increase in cell viability. The 3-(4,5-dimethylthiazol-2-yl)-2,5-diphenyltetrazolium bromide (MTT) assay evaluates the ability of viable cells to transform MTT into formazan crystals thanks to the intracellular reduction of MTT by oxidoreductase and dehydrogenase enzymes and electron donors [29].

As in rat cerebellum cultured neurons, Surin et al. [30] demonstrated that formazan formation occurs primarily in mitochondria; we could speculate that this increase in cell viability could be related to an enhancement in mitochondrial activity. Of course, we are aware that the MTT assay is more than a mere representation of mitochondrial activity [31] so, to demonstrate our hypothesis, further studies should be carried out.

### 2.3. Effects of ARs and EOs on LPS-Induced NO Production in BV-2 Cells

**Figure 2:**
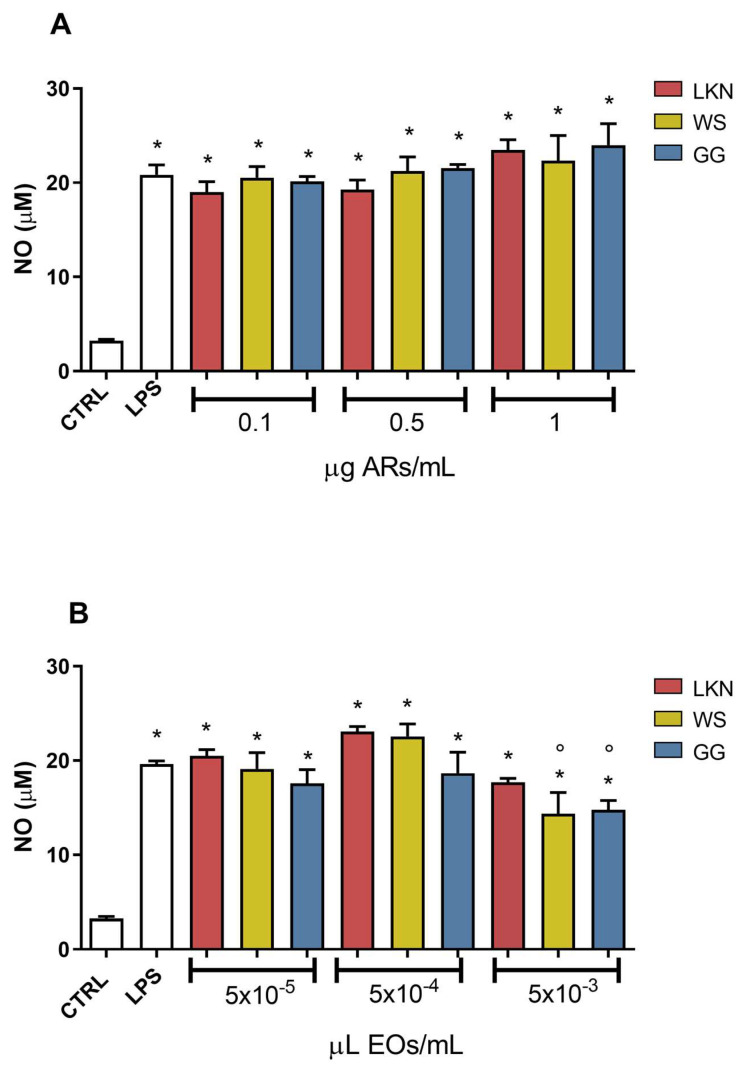
NO released by BV-2 cells treated with the different aqueous residues (ARs) and essential oils (EOs) and activated with LPS. BV-2 cells were pretreated for 2 h with increasing concentrations of White Shark (WS), Gorilla Glue (GG), and Lemon Conti Kush New (LKN) ARs (0.1–0.5–1 µg/mL) (**A**) and EOs (5 × 10^−5^–5 × 10^−4^–5 × 10^−3^ µL/mL) (**B**), and then exposed to 100 ng/mL LPS for a further 24 h. Each bar represents means ± SEM of at least three independent experiments. Data were analyzed by one-way ANOVA followed by Bonferroni’s test. * *p* < 0.05 vs. CTRL. ° *p* < 0.05 vs. LPS.

The potential anti-neuroinflammatory activity of the different ARs and EOs has been investigated by measuring the levels of nitric oxide (NO) released in the culture medium. BV-2 cells were treated with increasing concentrations of the three ARs or EOs for 2 h, and then activated with 100 ng/mL LPS. Cell culture medium was collected and analyzed for NO production using the Griess reagent. As expected, cells exposed to LPS significantly increased their release of NO with respect to control cells. Of note, none of the different ARs was able to prevent LPS-induced NO release at any tested concentration (Figure 2A).

On the other hand, at the lowest concentrations (5 × 10^−5^ and 5 × 10^−4^ µL/mL), none of the EOs was able to counteract LPS-induced NO production, while, at the highest concentration, only WS and GG significantly reduced LPS-induced NO production (Figure 2B). Based on the data obtained, only 5 × 10^−3^ µL/mL WS and GG EOs were selected for the subsequent experiments.

### 2.4. Effects of WS and GG EOs on Pro- and Anti-Inflammatory Mediators in LPS-Activated BV-2 Cells

**Figure 3:**
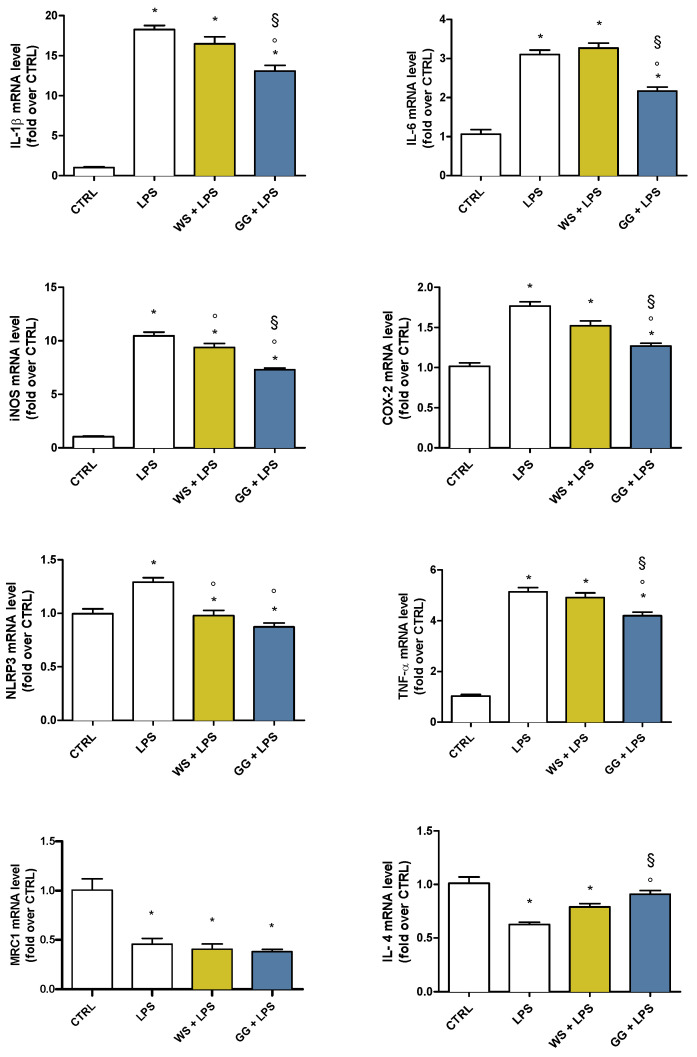
Expression of pro- and anti-inflammatory agents in BV-2 cells pretreated with WS and GG EOs and activated with LPS. BV-2 cells were pretreated with 5 × 10^−3^ µL/mL White Shark (WS) and Gorilla Glue (GG) EOs for 2 h and then exposed to 100 ng/mL LPS for a further 24 h. Gene expression was evaluated by RT-PCR as reported in Materials and Methods. Data are represented as fold-over CTRL. Each bar represents means ± SEM of at least three independent experiments. Data were analyzed by one-way ANOVA followed by Bonferroni’s test. * *p* < 0.05 with respect to CTRL; ° *p* < 0.05 with respect to LPS; § *p* < 0.05 with respect to WS + LPS.

To investigate the capability of WS and GG EOs to modulate pro- and anti-inflammatory mediators in activated BV-2 cells, cells were pretreated with 5 × 10^−3^ µL/mL WS and GG EOs for 2 h before undergoing LPS exposure for a further 24 h. At the end of each experiment, cells were lysed, and the RNA was collected to evaluate gene expression of IL-1β, TNF-α, IL-6, COX-2, iNOS, NLRP3, MRC1, and IL-4 by RT-PCR (Figure 3). Cells exposed to LPS significantly upregulated the pro-inflammatory genes (IL-1β, TNF-α, IL-6, COX-2, iNOS, and NLRP3), and downregulated the anti-inflammatory genes (IL-4 and MRC1), with respect to control cells. Only GG EO was able to significantly reduce the expression of all pro-inflammatory genes and to significantly increase the expression of the IL-4 anti-inflammatory gene with respect to LPS; on the other hand, WS EO significantly reduced iNOS and NLRP3 expression while showing no effect on the expression of both MRC1, and IL-4 anti-inflammatory genes. Interestingly, the expression of TNF-α, iNOS, COX-2, and IL-6 were significantly downregulated by GG EO compared with WS EO. Taken together, these data demonstrated that GG EO was more effective than WS EO in counteracting LPS-induced inflammatory status in BV-2 cells. For this reason, we decided to focus our attention only on GG EO in the next experiments.

### 2.5. GG EO Modulates iNOS, NLRP3, and COX-2 Protein Expression in LPS-Activated BV-2 Cells

**Figure 4:**
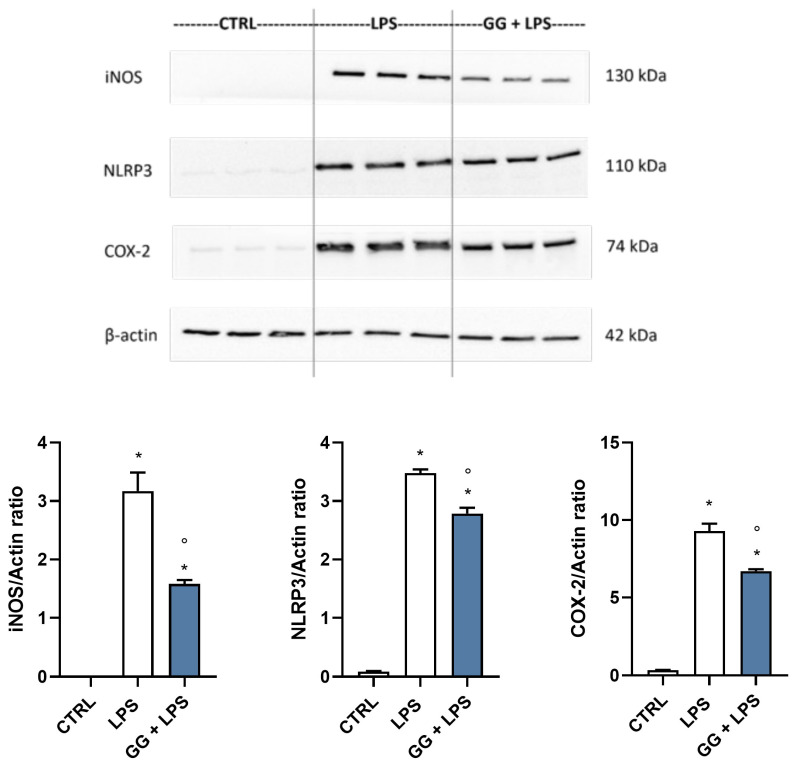
Protein expression of iNOS, NLRP3, and COX-2 in BV-2 cells exposed to GG EO before the activation with LPS. BV-2 cells were pretreated with 5 × 10^−3^ µL/mL Gorilla Glue (GG) EO for 2 h before the addition of 100 ng/mL LPS for a further 24 h. Immunoblotting was performed using anti-iNOS, anti-NLRP3, and anti-COX-2 specific antibodies. Each bar represents means ± SEM of three independent experiments. Data were analyzed by one-way ANOVA followed by Bonferroni’s test. * *p* < 0.05 with respect to CTRL; ° *p* < 0.05 with respect to LPS.

To further confirm the anti-inflammatory potential of GG EO, we explored the protein expression of the three main enzymes involved in the inflammatory process (iNOS, NLRP3, and COX-2) by Western Immunoblotting. BV-2 cells were seeded in 6-well plates and exposed to 5 × 10^−3^ µL/mL GG EO for 2 h before adding 100 ng/mL LPS for a further 24 h. In agreement with the RT-PCR results, LPS significantly increased the protein level of the three enzymes while the GG EO treatment was able to significantly reduce iNOS, NLRP3, and COX-2 protein level in respect to LPS-activated cells (Figure 4).

### 2.6. GG EO Counteracts NF-κB Nuclear Translocation

**Figure 5:**
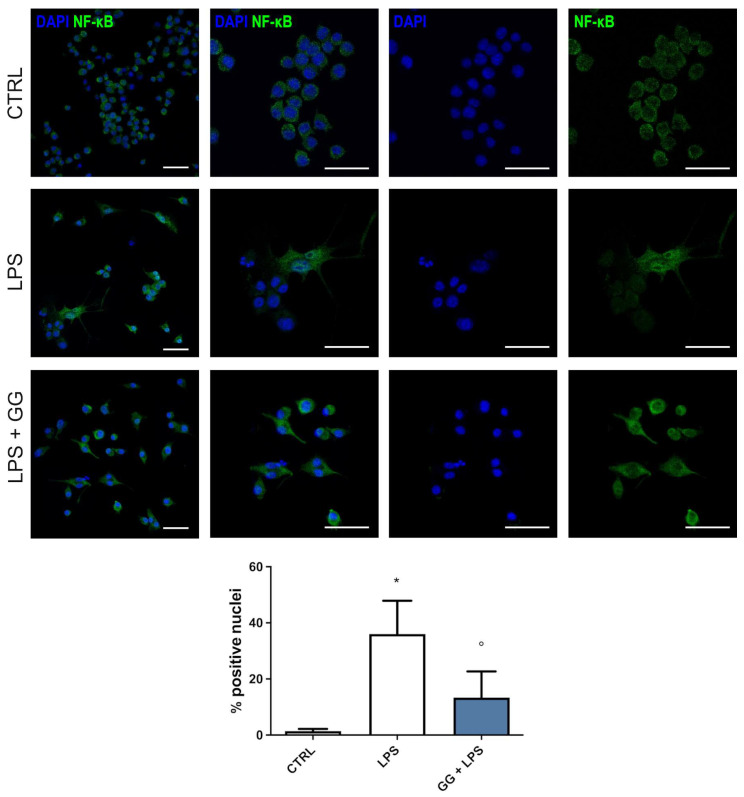
GG EO prevents NF-κB nuclear translocation. BV-2 cells were pretreated with 5 × 10^−3^ µL/mL Gorilla Glue (GG) EOs for 2 h and then exposed to 100 ng/mL LPS for a further 24 h. Cells were immunostained with a primary antibody against NF-κB, followed by a secondary Alexa Fluor 488-antibody (green), and cell nuclei (blue) were visualized with DAPI. Magnification images are shown both as single signals and as a merger. Scale bar = 50 µm. The graph shows the percentage of nuclei positive for NF-κB evaluated in five fields (around 100 cells) for each condition. * *p* < 0.05 vs. CTRL; ° *p* < 0.05 vs. LPS.

Nuclear factor kappa-light-chain-enhancer of activated B cells (NF-κB) transcription factor is a master regulator of the inflammatory signaling pathway, controlling the gene expression of pro-inflammatory cytokines and enzymes through its nuclear translocation. To unravel the mechanism beyond the reduction of LPS-induced upregulation of pro-inflammatory mediators elicited by GG EO, we evaluated the nuclear translocation of NF-κB transcription factor by immunofluorescence analysis. BV-2 cells were seeded on coverslips and treated with 5 × 10^−3^ µL/mL GG EO for 2 h, and then cells were stimulated with 100 ng/mL LPS for a further 24 h. As shown in Figure 5, the results clearly indicate a cytosolic localization of the NF-κB-positive signal in the control cells, and the differently NF-κB-positive signal is highly co-localized with paraformaldehyde, 4′-6-diamidino-2- phenylindole (DAPI) signal from nuclei in the LPS-treated cells, indicating a nuclear translocation of the protein. The pretreatment with GG EO was able to significantly reduce the NF-κB nuclear translocation, as the NF-κB-positive signal prevalently derives from a cytosolic localization.

### 2.7. Effect of GG EO on ROS and GSH Levels

**Figure 6:**
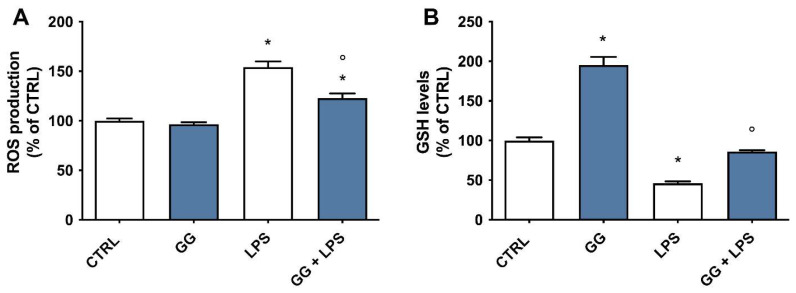
Antioxidant activity of GG EO in BV-2 cells activated with LPS. BV-2 cells were pretreated with 5 × 10^−3^ µL/mL Gorilla Glue (GG) EO for 2 h and then exposed or not to 100 ng/mL LPS for 24 h. (**A**)**.** Intracellular ROS levels were measured using the peroxide-sensitive probe DCFH-DA and (**B**)**.** GSH levels were measured using the fluorescent probe MCB. Each bar represents the mean ± SEM of three independent experiments. Data were analyzed by one-way ANOVA followed by Bonferroni’s test. * *p* < 0.05 with respect to CTRL; ° *p* < 0.05 with respect to LPS.

To study the potential protective effect of GG EO on LPS-induced alteration of redox status, we measured the levels of intracellular ROS and reduced glutathione (GSH) using specific probes like DCFH-DA and MCB, respectively (Figure 6). Cells were pretreated with 5 × 10^−3^ µL/mL GG EO for 2 h and then LPS was added or not for a further 24 h and ROS and GSH levels were evaluated by spectrofluorimetric analyses. As expected, LPS exposure induced a significant increase in the release of ROS with respect to control cells, meanwhile, the pretreatment with GG EO significantly prevented LPS-induced ROS production; GG EO treatment alone did not influence BV-2 redox state as ROS levels were comparable to those of control cells (Figure 6A). In agreement with the previous data, LPS significantly reduced intracellular GSH levels in respect to control cells. GG EO treatment, in the absence of LPS, significantly increased GSH levels in respect to control cells, suggesting an antioxidant effect of GG EO independently by neuro-inflammation. The ability of GG EO to boost GSH was also confirmed in cells activated with LPS, in which the treatment significantly increased GSH levels in respect to LPS-activated cells. (Figure 6B).

### 2.8. Modulation of p38 MAPK and Akt by GG EO

**Figure 7:**
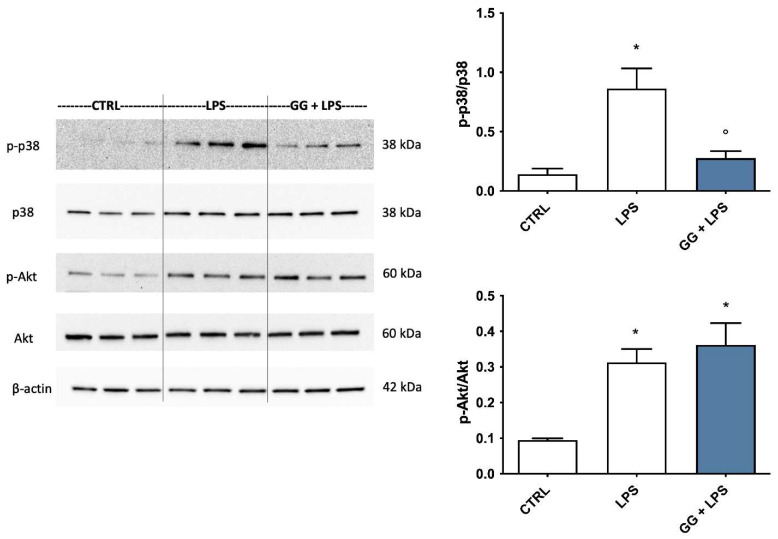
Effects of GG EO on p38 and Akt phosphorylation. BV-2 cells were pretreated with 5 × 10^−3^ µL/mL Gorilla Glue (GG) EO and then exposed to 100 ng/mL LPS for 24 h. Immunoblotting was performed using the total and the phosphorylated form of anti-p38 and anti-Akt specific antibodies. Each bar represents means ± SEM of three independent experiments. Data were analyzed by one-way ANOVA followed by Bonferroni’s test. * *p* < 0.05 vs. CTRL; ° *p* < 0.05 vs. LPS.

Mitogen-activated protein kinases (MAPKs), like p38, have been suggested to play a key role in the modulation of pro-inflammatory signaling pathways, and consequently in the production of inflammatory mediators in microglial cells [32]. At the same time, phosphoinositide 3-kinases/Akt (PI3K/Akt) signaling pathway is involved in the activation and nuclear translocation of NF-κB transcription factor [33]. Therefore, we investigated the effect of GG EO on LPS-induced phosphorylation of these two kinases (Figure 7). BV-2 cells were pretreated with 5 × 10^−3^ µL/mL GG EO and then exposed to 100 ng/mL LPS for another 24 h. LPS stimulation, as expected, induced a marked phosphorylation and thus activation of both proteins compared to untreated control cells, confirming the role of these two kinases in the pro-inflammatory stimuli. GG EO treatment showed a different effect on the two kinases. LPS-dependent p38 phosphorylation was totally inhibited by GG EO pretreatment, maintaining protein levels to values comparable with control cells, and suggesting a potential strong involvement of this kinases in the anti-inflammatory activity of GG EO. On the other hand, GG EO was not able to counteract the significant increase in Akt phosphorylation triggered by LPS, suggesting that this kinase could not be involved in GG EO anti-inflammatory activity.

### 2.9. Analysis of the Contribution of (E)-Caryophyllene, Caryophyllene Oxide, and Humulene to the Anti-Inflammatory Activity of GG EO

**Figure 8:**
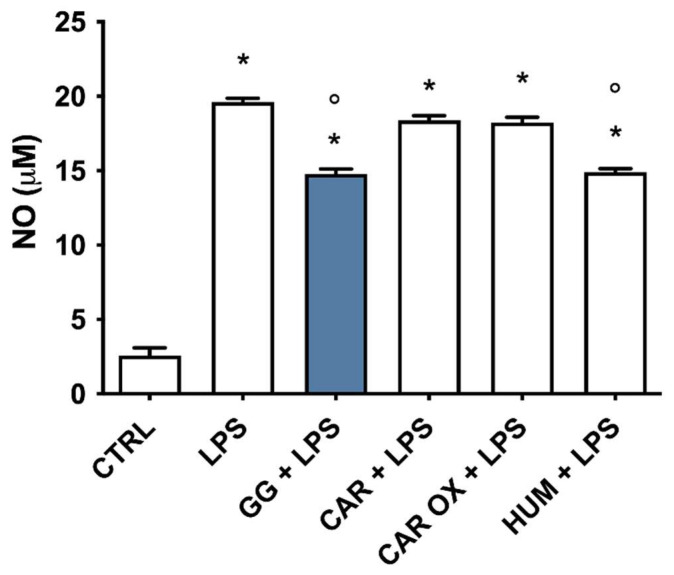
NO released by BV-2 cells pretreated with GG EO, CAR, CAR OX, and HUM and activated with LPS. BV-2 cells were pretreated for 2 h with 5 × 10^−3^ µL/mL Gorilla Glue (GG) EO, 4.14 µM (*E*)-caryophyllene (CAR), 1.35 µM of caryophyllene oxide (CAR OX), and 1.05 µM α-humulene (HUM), and then exposed to 100 ng/mL LPS for 24 h. Each bar represents means ± SEM of at least three independent experiments. Data were analyzed by one-way ANOVA followed by Bonferroni’s test. * *p* < 0.05 vs. CTRL; ° *p* < 0.05 vs. LPS.

Since GG EO compared with the other EOs contains higher levels of specific bioactive compounds, namely (E)-caryophyllene (CAR), caryophyllene oxide (CAR OX), and α-humulene (HUM), to unravel the potential anti-inflammatory contribution of these single components, we measured NO release in the culture medium as a biomarker of neuroinflammation. BV-2 cells were pretreated for 2 h with the same concentration of each compound present in 5 × 10^−3^ µL/mL of GG EO (4.14 µM CAR, 1.35 µM CAR OX, and 1.05 µM HUM) and then exposed to 100 ng/mL LPS for 24 h. Notably, only HUM induced a significant reduction in the release of NO with respect to LPS-exposed cells and its effect was comparable to that elicited by GG EO. On the contrary, neither CAR nor CAR OX were able to influence the release of NO induced by LPS (Figure 8).

**Figure 9:**
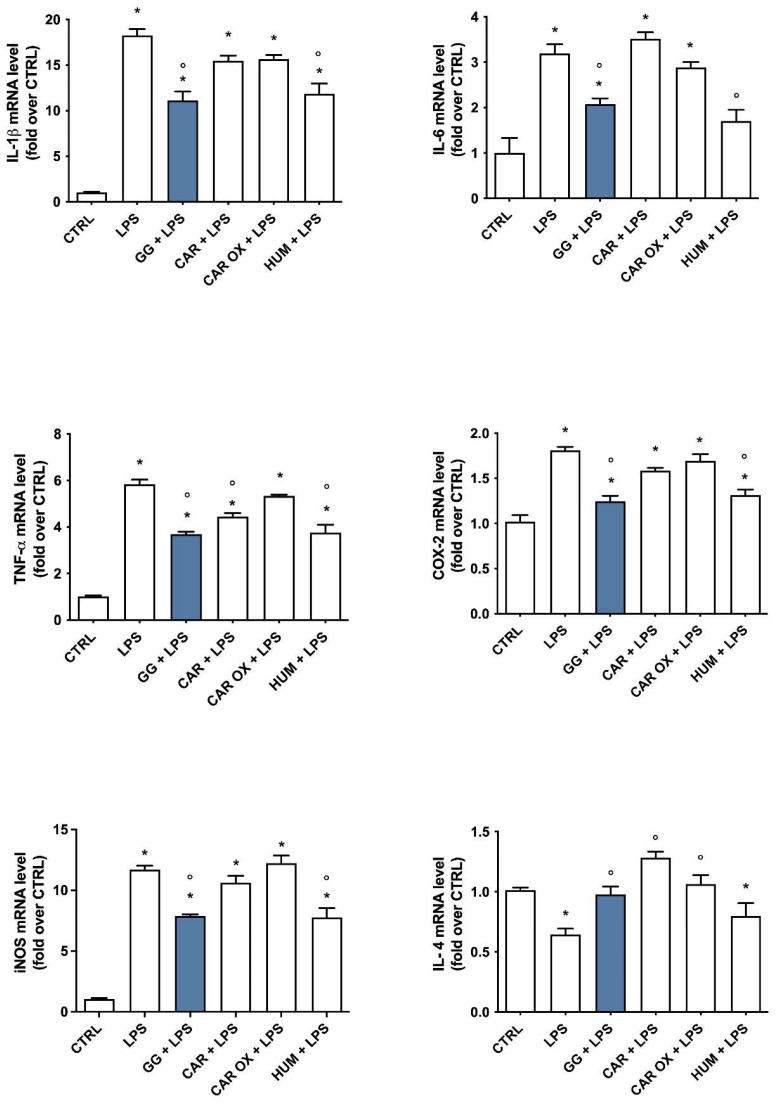
Expression of pro- and anti-inflammatory agents in BV-2 cells pretreated with GG EO, CAR, CAR OX, and HUM, and activated with LPS. BV-2 cells were pretreated with 5 × 10^−3^ µL/mL Gorilla Glue (GG) EO, 4.14 µM (*E*)-caryophyllene (CAR), 1.35 µM of caryophyllene oxide (CAR OX), and 1.05 µM µL/mL α-humulene (HUM) for 2 h and then they were exposed to 100 ng/mL LPS for a further 24 h. Gene expression of IL-1β, TNF-α, IL-6, COX-2, iNOS, and IL-4 was evaluated by RT-PCR. Data are represented as fold-over CTRL. Each bar represents means ± SEM of at least three independent experiments. Data were analyzed by one-way ANOVA followed by Bonferroni’s test. * *p* < 0.05 vs. CTRL; ° *p* < 0.05 vs. LPS.

In an effort to provide a more comprehensive characterization of the single contribution of CAR, CAR OX, and HUM to the anti-inflammatory activities shown by GG EO, we pretreated BV-2 cells with these compounds (4.14 µM, 1.35 µM, and 1.05 µM) for 2 h before the activation with 100 ng/mL LPS for a further 24 h. At the end of each experiment, RNA was extracted and the gene expression of IL-1β, TNF-α, IL-6, COX-2, iNOS, and IL-4 was analyzed by RT-PCR (Figure 9). Only GG EO and HUM significantly reduced LPS-induced upregulation of IL-1β, IL-6, COX-2, and iNOS gene expression, while GG EO, CAR, and HUM were able to significantly prevent the upregulation of TNF-α gene expression. Unexpectedly, CAR and CAR OX, which did not demonstrate any efficacy in inhibiting the pro-inflammatory cytokines, were instead capable of increasing the expression of the anti-inflammatory IL-4. Conversely, HUM did not influence this parameter, suggesting that HUM plays a role in modulating the M1 phenotype, while CAR and CAR OX trigger the transition to the M2 phenotype elicited by GG EO.

## 3. Discussion

Microglial activation plays a pivotal role in neuroinflammation, a common hallmark of neurodegenerative diseases [34,35]. In this context, a successful strategy should consider the use of therapeutic agents which act simultaneously on multiple mechanisms driving microglia activation. Natural products, and particularly *C. sativa*, owing to its abundance of phytochemical components, represent ideal candidates for counteracting neuroinflammation. Many studies have investigated the anti-neuroinflammatory activity of individual constituents of *C. sativa,* primarily THC and CBD, while the role of other *Cannabis* fractions (terpenes and flavonoids) in microglial activation remains poorly investigated. However, phenolic compounds found in *C. sativa* may interact with cannabinoids, modifying their action, a phenomenon referred to as the “entourage effect” [27]. Different studies have explored the synergistic effects of combinations of phytocannabinoids [36,37] as well as other bioactive secondary metabolites like terpenes and/or terpenoids [38,39]. For example, Ben-Shabat et al. observed that the endocannabinoid 2-arachidonoyl-glycerol shows enhanced activity in the presence of 2-acyl-glycerol esters, which are inactive on their own [40]. Another important aspect to consider is that the qualitative and quantitative composition of extracts obtained from hemp is strongly influenced by the hemp varieties [28,41]. Moreover, besides the intrinsic differences among varieties, it should be noted that the composition and bioactivities of extracts can be affected by the extraction method, solvent used, and temperature [28,42]. Therefore, the identification of specific varieties and appropriate extraction methods is crucial for the successful production of biologically active compounds from medicinal plants.

In this work, we elucidated the role of volatile terpenes and flavonoids from three commercial hemp varieties, obtained by hydrodistillation and hot water extraction, in the modulation of microglial inflammation. The rationale behind the choice of these varieties, which have previously been studied for their phytochemical composition [28], was their variance in terms of chemical profile.

BV-2 cells activated with LPS were used as a model of neuroinflammation. Previous studies have shown that LPS triggers gene expression of different inflammatory agents, such as TNF-α, IL-1β, IL-6, iNOS, and COX-2 as well as the production of NO and PGE2 in primary and BV-2 microglial cell cultures [43,44,45]. LPS is one of the most potent stimuli for microglial activation and has been widely used to mimic the inflammatory nature both in in vitro and in vivo models of neurodegenerative disorders [46]. Cells were pretreated with different concentrations of ARs and EOs for 2 h and then exposed to LPS for a further 24 h.

Our results on the bioactivity of the three ARs showed negligible effects in terms of anti-inflammatory activity as measured by the release of NO in the culture medium. On the other hand, GG and WS EOs significantly reduced the release of NO compared to LPS, indicating their potential activity against neuroinflammation. These results further confirm that the extraction methods can significantly impact the bioactivity of phytochemicals and each hemp variety possesses a specific profile of bioactivity that cannot not be generalized to all hemp varieties. Consistent with our findings, Orlando et al. [47] tested hemp EOs obtained from three different hemp varieties and only Futura 75 EO demonstrated the ability to inhibit Bcl-2 and TGFβ expression in human H1299 lung adenocarcinoma cells, while Carmagnola selezionata and Eletta campana EOs did not influence these parameters.

Similar to macrophages, microglia can polarize into either the M1 pro-inflammatory phenotype or M2 anti-inflammatory phenotype, depending on specific signals in the brain microenvironment [48]. Activated microglia stimulated by LPS are known to be neurotoxic, and are classified as M1-type microglia [49], resulting in the concomitant production and release of pro-inflammatory mediators such as iNOS, IL-1β, and TNF-α. These mediators trigger uncontrolled and chronic neuroinflammatory processes, inducing adjacent neuron impairment or death [50]. On the other hand, stimulation with IL-4 or IL-13 shifts microglia into the M2 anti-inflammatory phenotype. In this state, persisting microglial activation is able to promote neural tissue repair, neurogenesis, and anti-neuroinflammatory activity [51]. To better investigate the anti-inflammatory mechanisms underlined by WS and GG, the expression of different cytokines and inflammatory enzymes was evaluated. In agreement with the data on NO production, both EOs were able to significantly reduce iNOS expression, with GG being significantly more effective than WS. This higher efficacy of GG EO compared to WS EO was also observed in relation to other inflammatory agents, such as IL-1β, IL-6, COX-2, and TNF-α. Furthermore, GG EO significantly upregulated the anti-inflammatory interleukin IL-4 compared to both LPS and WS EO. As the two microglia-activated phenotypes can dynamically switch between each other, GG EO could enhance the neuroprotective activity of microglia by the switch to the M2 phenotype producing potential benefits against neurodegeneration.

Among the inflammatory mediators, NF-κB is a major transcription factor which effectively regulates the inflammatory processes by modulating the expression and production of various different pro-inflammatory proteins and enzymes, including IL-6, iNOS and COX-2 [52,53]. Moreover, several signaling pathways accompany NF-κB in modulating inflammatory events, including MAPKs like p38 MAPK, and phosphoinositide 3-kinases/Akt (PI3K/Akt) [54,55]. Among the MAPKs, p38 MAPK is considered the pivotal regulator of inflammation [56]. P38 MAPK is a class of MAPKs activated by stress stimuli such as inflammatory cytokines and ROS. Due to the high number of studies which have demonstrated the key role of p38 MAPK in chronic inflammation, preclinical or clinical trials for the application of p38 MAPK inhibitors in inflammatory diseases such as rheumatoid arthritis and asthma have been carried out [57]. P38 MAPK inhibitors, thanks to their ability to reduce inflammation, have recently been suggested as novel and potential therapeutic strategies to counteract neurodegenerative diseases. LPS activation induces a significant phosphorylation of p38 MAPK in BV-2 cells, as observed by many authors [58,59]. Furthermore, the Akt signaling pathway is involved in the pro-inflammatory activation triggered by LPS [60]. It has been observed that phosphorylated Akt triggers the upregulation of iNOS and COX-2 in LPS-activated BV-2 cells [61]. Accordingly, the identification of compounds targeting these signaling pathways is a promising approach for reducing uncontrolled inflammatory cascades in inflammation-associated diseases. The results of our study confirm that the anti-inflammatory activity of GG EO is associated with its ability to prevent the translocation to the nucleus of the pro-inflammatory transcription factor NF-kB and to strongly reduce p38 phosphorylation triggered by LPS. On the other hand, GG EO did not affect the LPS-stimulated phosphorylation of Akt, suggesting that Akt signaling was not involved in the inhibitory effects of GG EO on LPS-induced inflammatory responses in BV-2 cells.

Once activated, microglia produce ROS, which, in turn, can activate kinase cascades and transcription factors such as NF-κB, resulting in a vicious cycle which amplifies the neuroinflammatory processes and the subsequent neurodegeneration [62]. Notably, GG EO was able to reduce intracellular ROS production triggered by LPS and to increase the main endogenous antioxidant, GSH, suggesting that GG EO could exert both anti-inflammatory and antioxidant activity. The antioxidant potential of hemp EO has been previously observed by different authors [63,64,65] using in vitro antioxidant assays such as ABTS, DPPH assay, and FRAP assay.

The greater efficacy of GG compared to WS in counteracting neuroinflammation could be attributed to specific phytochemical compounds present in this EO. GG EO is, in fact, characterized by a greater presence of sesquiterpenes, such as CAR, CAR OX, and HUM compared to WS (Table 1) which is instead characterized by the presence of monoterpenes such as α- and β-pinene, myrcene and limonene. To verify our hypothesis, the anti-inflammatory activities of CAR, CAR OX, and HUM at the same concentrations present in GG EO were evaluated in LPS-activated BV-2 cells. Interestingly, our data show that among the three sesquiterpenes HUM was the main contributor to GG EO ability to reduce inflammatory mediators such as IL-1β, IL-6, TNF-α, COX-2, and iNOS, while CAR and CAR OX were able to increase the anti-inflammatory mediator IL-4’s contribution to the switch to the M2 phenotype. Interestingly, if CAR has been widely studied for its ability to affect the function of microglial cells, little or no evidence is available for CAR OX and HUM. Our data are partially in agreement with the results of Borgonetti et al. [22], who found CAR to be ineffective toward IL-1β, IL-6, and TNF-α upregulation in LPS-activated BV-2 cells. In our study, CAR did not influence IL-1β and IL-6 overexpression induced by LPS but was able to significantly reduce TNF-α. This difference in TNF-α modulation could be due to the different experimental conditions, like CAR concentration, length of pretreatment and LPS exposure. The ability of CAR to promote the M2 phenotype was also observed by Ascari et al. [66]. In particular, 1 μM (*E*)-caryophyllene induced a strong increase in the anti-inflammatory cytokine IL-10 in respect to LPS in primary murine microglia.

## 4. Materials and Methods

### 4.1. Chemicals

Dulbecco’s modified Eagle’s medium (DMEM), penicillin/streptomycin, L-glutamine solution, phosphate-buffered saline (PBS), MTT), 2′,7′-dichlorodihydrofluorescein diacetate (DCFH- DA), monochlorobimane (MCB), LPS from *Escherichia coli* serotype O127:B8, primers for real-time polymerase chain reactions (RT-PCR), RIPA buffer, sodium pyrophosphate, Triton X-100, paraformaldehyde, 4′-6-diamidino-2- phenylindole DAPI, dimethyl sulfoxide (DMSO), Griess reagent, sodium nitrite (NaNO_2_) and all other chemicals of the highest analytical grade were produced by Merck Italia (Milan, Italy). Low-endotoxin Fetal bovine serum (low-endotoxin FBS) was purchased from Euroclone (Milan, Italy). RNeasy Mini Kit was from Qiagen (Hilden, Germany). iScript^TM^ cDNA Synthesis Kit was purchased from Bio-Rad (Hercules, CA, USA). Excel-Taq FAST qPCR SybrGreen (no ROX) was purchased from SMOBIO Technology, Inc. (Hsinchu City, Taiwan). Standards of (*E*)-caryophyllene, α-humulene and caryophyllene oxide were purchased from Merk (Milan, Italy).

### 4.2. Plant Material and Extraction Processes

Pure dry inflorescences of the aforementioned hemp varieties, White Shark (WS), Gorilla Glue (GG), and Lemon Conti Kush New (LKN), were kindly provided by Everweed (https://www.everweed.it accessed on 23 July 2023) in September 2021. According to the work of Mazzara et al. [28], they were subjected to hydrodistillation for 5 h using a Clevenger-type apparatus and yielded 0.64–1.81% of yellowish EOs. After distillation, the aqueous residues in the reactor were collected and lyophilized to yield dry ARs. Chemical analyses of EOs and ARs were reported in Mazzara et al. [28]. Before biological assays, EOs and ARs were maintained at −20 °C and protected from light. As EOs are liquid oils their concentrations are expressed as μL/mL, while ARs are solid extracts and their concentrations are expressed as µg/mL.

### 4.3. Cell Culture and Treatments

Murine microglial cells (BV-2) were cultured in DMEM supplemented with 10% (*v*/*v*) of low-endotoxin FBS, 2 mM of L-glutamine, 50 U/mL of penicillin, and 50 μg/mL of streptomycin, and maintained at 37 °C in a humidified incubator with 5% CO_2_ as reported in [67]. BV-2 cells were kindly provided by Prof. Elisabetta Blasi of the University of Modena and Reggio Emilia, Italy. BV-2 microglial cells were pretreated for 2 h with different concentrations (5 × 10^−3^, 5 × 10^−4^ and 5 × 10^−5^ μL/mL), (0.1, 0.5, 1 µg/mL) of 3 different *C. sativa* EOs and ARs (LC, WS, and GG), respectively, or 4.14 µM of (*E*)-caryophyllene (CAR), 1.35 µM of caryophyllene oxide (CAR OX), and 1.05 µM of α-humulene (HUM), then LPS was added for a further 24 h to obtain a final concentration of 100 ng/mL. Cells were used for experiments from passages 15 to 20.

### 4.4. Viability Assay

MTT assay was used to evaluate cell viability, as previously reported [68]. BV-2 microglial cells were exposed for 24 h to different concentrations (5 × 10^−2^, 5 × 10^−3^ and 5 × 10^−4^ μL/mL), (1, 10, 50, 100 µg/mL) of 3 different *C. sativa* EOs and ARs (LC, WS, and GG), respectively. At the end of each experiment, cells were incubated with MTT work solution (0.5 mg/mL) for 30 min at 37 °C and then the MTT solutions were replaced with 100 μL of DMSO to dissolve the formed formazan crystals. A multilabel plate spectrophotometer (VICTOR3 V Multilabel Counter; PerkinElmer, Wellesley, MA, USA) was used to measure the absorbance at a wavelength of 595 nm. Cell viability was expressed as % of control cells.

### 4.5. Nitric Oxide Production

Microglial production of NO was evaluated by measuring the levels of nitrite released into culture media using the Griess reagent. BV-2 cells were seeded into 24-well culture plates at the density of 4.2 × 10^4^ cell/well. At the end of the experiments, cell media were collected, centrifuged at 1200 rpm for 5 min at 4 °C and the resulting supernatant was used to measure NO levels. Fifty μL of supernatant from each sample were added to the same volume of Griess reagent in a 96-well assay plate and after 15 min the absorbance was measured at 540 nm using a multilabel plate spectrophotometer (VICTOR3 V Multilabel Counter; PerkinElmer, Wellesley, MA, USA). Nitrite concentration was calculated with reference to a NaNO_2_ standard curve generated with known concentrations.

### 4.6. Measurement of Intracellular ROS Levels

BV-2 microglial cells were pretreated for 2 h with GG EO at a concentration of 5 × 10^−3^ μL/mL and then LPS was added or not for a further 24 h to obtain a final concentration of 100 ng/mL. At the end of the experiments, a DCFH-DA fluorescent probe was used to evaluate the intracellular ROS levels, as previously reported [69]. At the end of the treatments, a solution of 10 μM of DCFH-DA (DMEM w/o FBS and phenol red) was added to the BV-2 cells and incubated for 30 min at 37 °C. After this period, the probe was replaced with PBS. Cell fluorescence was measured at 485 nm (excitation) and 535 nm (emission) with a multilabel plate reader (VICTOR3 V Multilabel Counter; PerkinElmer, Wellesley, MA, USA). Intracellular ROS levels were expressed as % of control cells.

### 4.7. Measurement of Reduced GSH Levels

BV-2 microglial cells were pretreated for 2 h with GG EO at a concentration of 5 × 10^−3^ μL/mL and then LPS was added or not for a further 24 h to obtain a final concentration of 100 ng/mL. After the treatments, GSH levels were determined using the monochlorobimane (MCB) fluorometric assay as previously reported [70]. At the end of the experiments, cells were incubated with 50 μM MCB (DMEM w/o FBS and phenol red) for 30 min at 37 °C and then the probe was replaced with PBS. The fluorescence was measured at 355 nm (excitation) and 460 nm (emission) with a multilabel plate reader (VICTOR3 V Multilabel Counter; PerkinElmer).

### 4.8. Real-Time Polymerase Chain Reaction (PCR)

To evaluate the anti-inflammatory activities, BV-2 microglial cells were pretreated for 2 h with GG or WS EOs at the concentration of 5 × 10^−3^ μL/mL, or 4.14 µM of CAR, 1.35 µM of CAR OX, and 1.05 µM of HUM, and then LPS was added for a further 24 h to obtain a final concentration of 100 ng/mL. A RNeasy Mini Kit (Qiagen, GmbH, Hilden, Germany) was used to extract total RNA from each sample, following the manufacturer’s protocol, and the yield and purity of RNA were evaluated employing a NanoVue spectrophotometer (GE Healthcare, Milan, Italy). cDNA was obtained by reverse-transcribing 1 μg of total RNA using an iScript cDNA Synthesis Kit (BIO-RAD, Hercules, CA, USA), following the manufacturer’s protocol. Subsequently, PCR was carried out in a total volume of 10 μL (2.5 μL (12.5 ng) of cDNA, 5 μL Excel-Taq FAST qPCR SybrGreen (SMOBIO Technology, Inc. Hsinchu City, Taiwan), and 0.4 μL (400 nM) of each primer), as previously reported [71]. The primers used are reported in Table 2. Initially, the polymerase was activated for 30 s at 95 °C, and then the cDNA amplification was carried out through 40 cycles of 5 s at 95 °C and 30 s at 60 ◦C. GAPDH was used as a reference gene for BV-2 cells. To ensure quality control and generation of a single product, melt curves were run. According to the 2^−∆∆CT^ method [72], normalized expression levels were calculated with respect to control cells.

### 4.9. Western Immunoblotting

BV-2 microglial cells were pretreated for 2 h with GG EO at the concentration of 5 × 10^−3^ μL/mL and then LPS was added for further 24 h to obtain a final concentration of 100 ng/mL. Ice-cold PBS was used to wash cells and a RIPA buffer solution with mammalian protease inhibitor cocktail (1:100 dilution), 10 mg/mL phenylmethylsulfonyl fluoride, and PhosSTOP 1X (Roche, Mannheim, Germany) were used to lyse cells. Samples were boiled for 5 min at 96 °C, and then separated on 4–20% SDS-polyacrylamide gels (20 μg/lane) (BIO-RAD, Hercules, CA, USA). Proteins were transferred using a nitrocellulose membrane (BIO-RAD, Hercules, CA, USA) in Tris-glycine buffer at 110 V for 90 min. After 5 min incubation in blocking buffer (EveryBlot Blocking Buffer, BIO-RAD, Hercules, CA, USA), membranes were incubated with anti-NLRP3 (#15101), anti-iNOS (#13120), anti-Cox2 (#12282), anti-p38 (#9212), anti-p-p38 (#9211), anti-Akt (#9272), anti-p-Akt (#9271) (Cell Signaling Technology, Beverly, MA) (1:1000 dilution) and anti-β-actin (A5441, Sigma Aldrich–Merck) (1:5000 dilution) as internal loading control, overnight at 4 °C on a 3D rocking table. ClarityTM Western ECL Substrate (BIO-RAD, Hercules, CA, USA) was used to visualize marked proteins. Densitometric analysis of specific immunolabeled bands was performed using ImageLab software version 5.2.

### 4.10. Immunofluorescence Confocal Microscopy

BV-2 cells were seeded directly on glass coverslips in 6-well plates and, at the end of treatments, were fixed with paraformaldehyde 2% for 15 min at room temperature. Fixed cells were permeabilized with Triton X-100 0.1% for 10 min. Subsequently, BV-2 cells were incubated overnight with a polyclonal antibody (1:500) against NF-κB p65 (Sigma-Aldrich-Merck) and then with a secondary Alexa Fluor 488-conjugated antirabbit IgG antibody (1:1000) (Life Technologies Italia, Monza, MB, Italy) for 1 h at room temperature. Nuclei were stained using 4′-6-diamidino-2-phenylindole (DAPI) 1 μg/mL for 10 min. Confocal imaging was performed using a Nikon A1 confocal laser scanning microscope, as previously described [73]. The confocal serial sections were processed with ImageJ software (https://imagej.net/ij/) to obtain three-dimensional projections. The image rendering was performed by Adobe Photoshop software (https://helpx.adobe.com/). The percentage of nuclei positive for NF-kB was evaluated by two operators in five fields (around 100 cells) for each condition.

### 4.11. Statistical Analysis

Each experiment was performed at least three times, and all values are represented as mean ± SEM. One-way analysis of variance (ANOVA) was used to compare differences among groups, followed by Dunnett’s or Bonferroni’s test (GraphPad Prism 9.4.1, San Diego, CA, USA). Values of *p* < 0.05 were considered statistically significant.

## 5. Conclusions

Our work showed that EOs from industrial *C. sativa* are more effective than ARs, and among the EOs only those characterized by high levels of sesquiterpenes seem to be promising candidates for the treatment of neuroinflammation. In this respect, our results evidenced a major contribution of HUM in the anti-neuroinflammatory activity displayed by GG EO. To our knowledge, this is the first time that the anti-inflammatory activity of HUM has been observed in microglial cells. However, it is important to note that the data presented here will need to be confirmed through more comprehensive studies to better understand its true effectiveness and mechanisms of action in the context of neuroinflammation. Another important aspect evidenced by our study is that the use of EOs, with their complex pattern of phytochemicals, can evoke a pleiotropic action through the synergistic contribution of different components, as opposed to a single compound.

## Figures and Tables

**Table 1:** Main components detected in the 3 commercial varieties’ EOs and ARs.

Compound	Hemp Varieties		
	White Shark	Lemon Conti Kush New	Gorilla Glue
EO		(g/100 g)	
α-pinene	8.53	3.24	0.74
β-pinene	3.59	2.78	0.82
myrcene	20.28	11.14	7.16
limonene	9.17	5.97	6.79
terpinolene	9.51	30.47	0.27
(*E*)-caryophyllene	10.79	10.40	18.94
humulene	2.96	3.04	4.86
caryophyllene oxide	3.52	3.34	6.62
CBD	5.54	2.91	3.06
AR	(g/100 g)		
cannabisin B	0.71	0.35	0.30
luteolin-C-hexoside-O-rutinoside	0.23	0.50	2.51
rutin	7.93	0.35	0.75
luteolin-hexoside-hexoside	0.21	2.35	0.21
apigenin-hexoside-glucuronide	1.12	0.32	0.79

**Table 2:** Primers for RT-PCR in BV-2 cells.

Gene	5′-Forward-3′	5′-Reverse-3′
*IL-1β*	GTTCCCATTAGACAACTGCACTACAG	GTCGTTGCTTGGTTCTCCTTGTA
*TNF-α*	CCCCAAAGGGATGAGAAGTTC	CCTCCACTTGGTGGTTTGCT
*iNOS*	CCTCCTCCACCCTACCAAGT	CACCCAAAGTGCTTCAGTCA
*COX-2*	TGGGGTGATGAGCAACTATT	AAGGAGCTCTGGGTCAAACT
*Nlrp3*	GATGCTGGAATTAGACAACTG	GTACATTTCACCCAACTGTAG
*IL-6*	TCCTTCAGAGAGATACAGAAAC	TTCTGTGACTCCAGCTTATC
*IL-4*	CTGGATTCATCGATAAGCTG	TTTGCATGATGCTCTTTAGG
*MRC1*	GTTATGAAAGGCAAGGATGG	ATCAGTGAAGGTGGATAGAG
*GAPDH*	ACCACAGTCCATGCCATCAC	TCCACCACCCTGTTGCTGTA

## Data Availability

The data are available on request from the authors.
